# Blinding of outcome assessors and its association with outcome in a randomized open-label stroke trial

**DOI:** 10.1177/17474930221131706

**Published:** 2022-10-19

**Authors:** Nadinda AM van der Ende, Bob Roozenbeek, Joseph P Broderick, Pooja Khatri, Hester F Lingsma, Diederik WJ Dippel

**Affiliations:** 1 Department of Neurology, Erasmus MC University Medical Center, Rotterdam, The Netherlands; 2 Department of Radiology and Nuclear Medicine, Erasmus MC University Medical Center, Rotterdam, The Netherlands; 3 Department of Neurology and Rehabilitation Medicine, University of Cincinnati Gardner Neuroscience Institute, University of Cincinnati Academic Health Center, Cincinnati, OH, USA; 4 Department of Public Health, Erasmus MC University Medical Center, Rotterdam, The Netherlands

**Keywords:** Clinical trial, ischemic stroke, acute stroke therapy, intervention, methodology, neurology

## Abstract

**Background:**

It is challenging for outcome assessors to remain blinded during outcome assessment in trials with prospective randomized open blinded endpoint (PROBE) design. If assessors are able to guess the correct treatment allocation more often than expected based on chance, the assessors may have been not properly blinded.

**Aims:**

We aimed to assess blinding of outcome assessors in a stroke trial with PROBE design and its association with outcome.

**Methods:**

We used data of the Interventional Management of Stroke (IMS) III trial. The modified Rankin Scale (mRS) at 90 days was assessed by local assessors who were unaware of treatment allocation. To assess success of blinding, each assessor was asked to guess the patient’s treatment allocation. We assessed whether the percentage of correct guesses was higher than chance (i.e. 50%). The association between correctly guessed treatment allocation and the mRS at 90 days was analyzed with ordinal logistic regression stratified by treatment allocation. We tested for interaction of correctly versus incorrectly guessed treatment allocation with actual treatment allocation on the mRS. Patients with missing data on guessed treatment allocation and patients who died prior to 90-day assessment were excluded.

**Results:**

In total, 459 patients were included in this study. The assessors guessed the correct treatment allocation significantly more often than expected (267/459, 58.2%, one-sided p = 0.0003). Correctly guessed treatment allocations were associated with better mRS scores in the intervention group (common odds ratio (cOR): 2.28, 95% confidence interval (CI): 1.50–3.48) and with worse mRS scores in the control group (cOR: 0.47, 95% CI: 0.27–0.83) (p_interaction_ < 0.001).

**Conclusions:**

Assessors may not always be truly blinded for treatment allocation in clinical trials, and their guesses may be associated with outcome. Although causality between the assessors’ guess and patient outcome cannot be determined, future trials with subjective outcome should make efforts to ensure blinding and should report their blinding method and the success of blinding like the IMS III trial.

**Clinical Trial Registration:**

URL: https://clinicaltrials.gov. Unique identifier: NCT00359424.

## Introduction

Blinding is important to minimize bias in outcome assessment of clinical trials, particularly in trials with subjective outcomes.^1,2^ If outcome assessment is not properly blinded for treatment allocation, this may lead to biased treatment effect estimates.^3,4^

In trials with a prospective randomized open blinded endpoint (PROBE) design, patients and their proxies are aware of the treatment they received. Therefore, it may be challenging for outcome assessors to remain blinded if outcome assessment is conducted in person or by telephone (i.e. interactive outcome assessment). If assessors are able to guess the correct treatment allocation more often than expected based on chance, the assessors may have been not properly blinded.^5^

Many trials do not collect information about inadvertent unblinding by patients and proxies or by inadvertent review of other data sources, and, even if collected, trials do not report this information.^6–8^ We aimed to assess blinding of outcome assessors in a stroke trial with PROBE design and its association with outcome.

## Methods

### Data

We used data from the Interventional Management of Stroke (IMS) III trial.^9,10^ In short, IMS III was a phase 3, multicenter, clinical trial with PROBE design that evaluated the efficacy and safety of endovascular treatment plus intravenous thrombolysis in a dose of 0.6 mg/kg (intervention) compared to intravenous thrombolysis alone (control). The IMS III trial enrolled 656 patients from 58 international centers between August 2006 and April 2012, aged 18 to 80 years with a moderate-to-severe ischemic stroke (National Institutes of Health Stroke Scale ⩾ 10) before initiation of intravenous thrombolysis. Patients were randomly assigned in a 2:1 ratio to the intervention group or control group. The primary outcome was a modified Rankin Scale (mRS) score of 2 or less at 90 days. The mRS is a measure of functional outcome, which evaluates the degree of disability or dependence in daily life, and ranges from 0 (no symptoms) to 6, with 5 indicating severe disability and 6 indicating death. A score of 2 or less implies functional independence.^11^ The trial was approved by the ethics committee and research board of each participating center. Written informed consent was obtained from patients or their legal representative before enrollment in the trial.

### Clinical outcome assessment

Clinical outcome assessment at 90 days was performed by study investigators (assessors) who were not directly involved with acute treatment of the patient and who were unaware of treatment assignment. Patients were instructed not to discuss their initial hospitalization and treatment. Each assessor, as the final act of blinded assessment, guessed whether they believed the subject was in the intervention group or control group at the end of outcome assessment. In addition, they indicated how sure they were of their answer and listed items that their guess was based upon. They were not specifically asked if patients or proxies inadvertently mentioned information about the procedure. However, as this is a protocol violation, this should have been reported to the trial coordinators. The questionnaire is shown in Supplemental Table I.

### Statistical analysis

The trial was analyzed according to the intention-to-treat principle. For this study, patients without guessed treatment allocation (i.e. missing data) by the outcome assessor and patients who died prior to 90-day assessment were excluded. Clinical characteristics of patients included in this study were compared with patients who were excluded because of missing indication of treatment allocation. We described the degree (i.e. number of patients and percentage) of correctly guessed treatment allocations in the total population and stratified by treatment allocation. Whether the percentage of correct guesses was higher than chance (i.e. 50%) was assessed with a binomial probability test (one-sided p value). The difference in the degree of correctly guessed treatment allocations over treatment groups was compared with a chi-square test. We also describe the degree of correctly guessed treatment allocations according to how sure the assessors were of their guess. Differences in the frequencies of how sure the assessors were of their guess between the intervention group versus control group were compared with a Fisher’s exact test. Items that the assessors based their guess upon were described by guessed treatment allocation by the assessor.

The association between correctly guessed treatment allocation and the mRS at 90 days was analyzed with ordinal logistic regression stratified by actual treatment allocation and is presented as common odds ratio (cOR) with 95% confidence interval (CI) to indicate statistical precision. We used a multiplicative interaction term to test for interaction of correctly versus incorrectly guessed treatment allocation with actual treatment allocation on the mRS. Statistical analyses were performed with Stata/SE 16.1 (StataCorp, Texas, USA).

## Results

**Figure 1.**
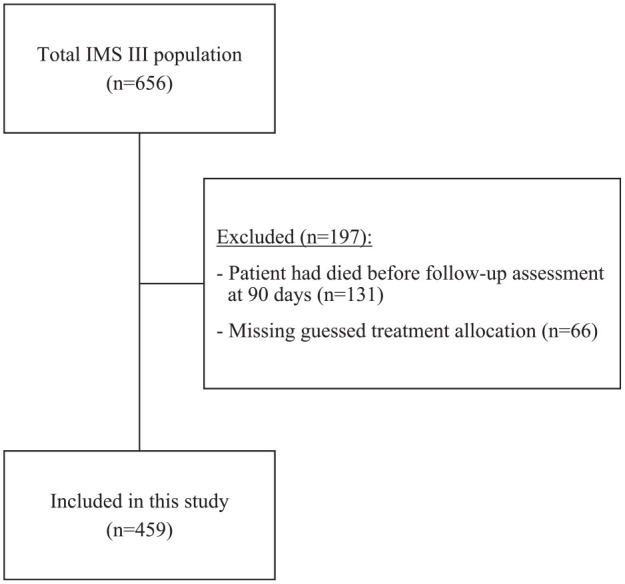
Flowchart of patients included in this study. IMS: Interventional Management of Stroke.

Of the 656 patients included in the IMS III trial, 131 patients could not be assessed within 90 days due to prior death. Among the remaining 525 patients, 459 (87.4%) patients had data on the blinded assessor’s designation of treatment allocation and were included in this study (Figure 1). The median age was 67 (interquartile range (IQR): 56–75) years, 238 (51.9%) patients were men, baseline National Institutes of Health Stroke Scale was 16 (IQR: 13–20), and 304 (66.2%) patients were randomized to the intervention group. Patients included in the analyses showed similar baseline clinical characteristics, treatment assignment, and 90-day clinical outcomes, as patients were excluded because their treatment allocation was not guessed by the assessor (Table 1).

**Table 1.** Clinical characteristics of patients included in this study versus excluded patients with unknown guess of treatment allocation.

	Included patients (n = 459)	Excluded patients with missing guess of treatment allocation (n = 66)
Age in years—median (IQR)	67 (56–75)	68 (54–75)
Men—n (%)	238 (51.9)	30 (45.5)
Baseline NIHSS—median (IQR)*	16 (13–20)	15 (12–17)
Time from symptom onset to randomization in min—median (IQR)	143 (118–170)	142 (125–156)
Randomized to the intervention group—n (%)	304 (66.2)	47 (71.2)
Modified Rankin Scale at 90 days—median (IQR)^†^	2 (1–4)	2 (1–4)

IQR: interquartile range presented as the 25th and 75th percentile; NIHSS: National Institutes of Health Stroke Scale.

* Baseline NIHSS score was missing for 1/459 (0.2%) patient in the group of included patients.

† Modified Rankin Scale score at 90 days missing for 13/66 (19.7%) patients in the group of excluded patients with missing guess of treatment allocation.

### Guess of treatment allocation by the assessors

The assessors guessed the correct treatment allocation significantly more often than expected (267/459, 58.2%, one-sided p = 0.0003) (Table 2). The assessors guessed the treatment allocation correctly in 183/304 (60.2%) patients in the intervention group and in 84/155 (54.2%) patients in the control group. There was no difference in percentage of correctly guessed treatment allocations between treatment groups (p = 0.218). The percentage of correctly guessed treatment allocations increased when the assessors were more sure of their guess (Table 3). The percentage of correctly guessed treatment allocations was equal to chance if the assessors indicated that they were not sure of their guess (p = 0.146).

**Table 2.** Correctly guessed treatment allocations by the assessors in the total cohort and stratified by actual treatment allocation.

	Total (n = 459)	Intervention group (n = 304)	Control group (n = 155)
Correctly guessed treatment allocation—n (%)	267 (58.2)	183 (60.2)	84 (54.2)
Incorrectly guessed treatment allocation—n (%)	192 (41.8)	121 (39.8)	71 (45.8)
p value	0.0003*	0.218^†^	

* The p value for more than 50% correctly guessed treatment allocations (one-sided binomial probability test).

† Comparison of intervention group versus control group.

**Table 3.** Correctly guessed treatment allocations by the assessors according to how sure the assessors were of their guess in the total cohort and stratified by treatment allocation.

	Total (n = 459)			Intervention group (n = 304)			Control group (n = 155)		
	Very sure (n = 28)	Somewhat sure (n = 105)	Not sure (n = 326)	Very sure (n = 24)	Somewhat sure (n = 67)	Not sure (n = 213)	Very sure (n = 4)	Somewhat sure (n = 38)	Not sure (n = 113)
Correctly guessed treatment allocation—n (%)	24 (85.7)	70 (66.7)	173 (53.1)	21 (87.5)	47 (70.1)	115 (54.0)	3 (75.0)	23 (60.5)	58 (51.3)
Incorrectly guessed treatment allocation—n (%)	4 (14.3)	35 (33.3)	153 (46.9)	3 (12.5)	20 (29.9)	98 (46.0)	1 (25.0)	15 (39.5)	55 (48.7)
p value	0.0001*	0.0004*	0.146*			0.065^†^			

* The p value for more than 50% correctly guessed treatment allocations (one-sided binomial probability test).

† Comparison of intervention group versus control group.

### Items that the assessors based their guess upon

**Figure 2.**
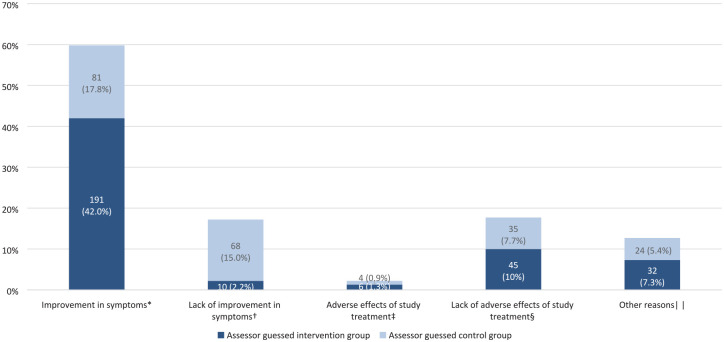
Items that the assessors based their guess upon and the guessed treatment allocation by the assessors. ^*^Data on improvement in symptoms were missing for four patients. ^†^Data on lack of improvement in symptoms were missing for six patients. ^‡^Data on adverse effects of study treatment were missing for six patients. ^§^Data on lack of adverse effects of study treatment were missing for seven patients. ^||^Data on other reasons were missing for 18 patients.

The assessors mostly based their guess on improvement in symptoms (n = 272, 59.8%, Figure 2), and this was more often used to guess the intervention group (42% vs. 17.8%, p < 0.001). Lack of improvement was more often used to guess the control group (15% vs. 2.2%, p < 0.001).

### Association between correctly guessed treatment allocation and outcome

**Figure 3.**
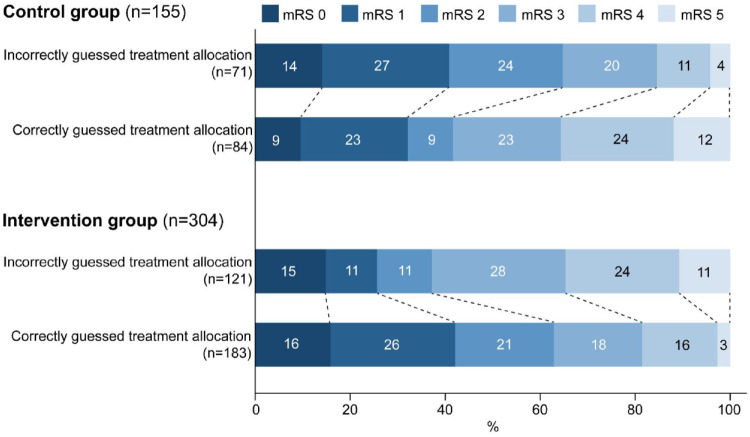
Functional outcome by incorrectly versus correctly guessed treatment allocation by the assessor stratified by actual treatment allocation. Correctly guessed treatment allocations were associated with better mRS scores in the intervention group (cOR: 2.28, 95% CI: 1.50–3.48) and with worse mRS scores in the control group (cOR: 0.47, 95% CI: 0.27–0.83) (p_interaction_ < 0.001). mRS: modified Rankin Scale.

There was an interaction between correctly versus incorrectly guessed treatment allocation and the actual treatment allocation on the mRS (p_intraction_ < 0.001); correctly guessed treatment allocations were associated with better mRS scores in the intervention group (cOR: 2.28, 95% CI: 1.50–3.48) and with worse mRS scores in the control group (cOR: 0.47, 95% CI: 0.27–0.83; Figure 3).

## Discussion

In this study, we assessed blinding of outcome assessors in a stroke trial with PROBE design and its association with the mRS, which is a relatively subjective outcome. Our study suggests that assessors might not be truly blinded for treatment allocation in trials with PROBE design and that their guessed treatment allocation is associated with outcomes.

In trials with PROBE design, patients and health care providers are not blinded, but outcome assessors are assumed to be blinded.^12^ This assumption might not be true for all trials with PROBE design, as we found that outcome assessors were able to guess the correct treatment allocation more often than expected based on chance. Therefore, it is important that the possibility of unblinding of outcome assessors to treatment allocation is considered and is assessed and reported, as was uniquely done in the IMS III trial.

Our finding that outcome assessors were able to guess the correct treatment allocation more often than expected based on chance could be explained by several reasons. It could be explained by the use of local assessors. Local assessors, compared to central assessors, might be more at risk for unblinding, because local assessors can (unintentionally) be informed about treatment allocation during the patient’s hospital admission and they can have access to information related to treatment allocation. Another explanation is that the assessors’ guess of treatment allocation might be influenced by outcomes (i.e. reversed causality). Although causality of our finding cannot be determined, researchers and clinicians should pay attention to the possibility of unblinding of outcome assessors in trials with PROBE design, especially since the outcome assessment method as in the IMS III trial is still used in clinical trials with PROBE design.^13^ Central outcome assessors should be considered as they decrease the likelihood of unblinding, because they cannot (unintentionally) be informed about treatment allocation during the patient’s hospital admission and they do not have access to other sources of information related to treatment allocation.

Outcome assessors indicated that they based their guess of treatment allocation often on improvement or lack of improvement. Although this follows logic, it is important to realize that outcome assessors might associate outcomes or side effects with intervention, which can also be the case in double-blinded trials.

Success of blinding of outcome assessors is often not assessed and/or reported.^6,7^ Because unblinding may lead to biased treatment effect estimates,^3,4^ we recommend that the methods and effectiveness of blinding in trials with subjective outcome should be assessed and reported, for example, by adding this item to the CONSORT (Consolidated Standards of Reporting Trial) checklist and the SPIRIT (Standard Protocol Items: Recommendations for Interventional Trials) guideline.^14,15^ Assessment of the blind should include for assessors to make a forced choice between treatment arms.^5^ It is important to present only the treatment arms (e.g. “control” and “intervention”) as alternatives, because introducing the possibility of answering with the socially correct “I do not know” makes the results of your test difficult to interpret. To evaluate the influence of outcome assessment on the assessors’ guess, the assessors should be asked to guess the treatment allocation both prior to and after assessment. In addition, it should be assessed whether patients or their proxies brought up information related to treatment to the blinded assessor. For example, by including a question in which the assessor should indicate if the patient or their proxy had brought up this information or perhaps better by recording outcome assessment and to verify whether patients or proxies had brought up information about the treatment. Furthermore, as the preconceived belief of the assessor might influence outcome assessment, measuring the beliefs about intervention efficacy and safety during the trial—as these beliefs may change during the trial—can be informative to help understand the relevance of (in)correct guesses of treatment allocation.^16^ Alternatively, to validate outcomes and to assure blinded outcome assessment, the use of external, blinded outcome adjudication can be beneficial and should be considered.^17^ For example, by the use of an outcome committee that adjudicates masked reports based on structured interviews of the assessors. Finally, we emphasize that everyone who relies on the results of randomized trials should consider the risk of unblinding.

This study has several limitations. First, the assessors were asked to guess what treatment group the patient was assigned to, but we do not know if the patient or their proxy brought up information related to treatment during the interview, although this should have been reported to the trial coordinators as a protocol violation, and none of these reports occurred. It cannot be fully known whether assessors were truly unblinded or that the outcome affected the assessors’ guess of treatment allocation (i.e. reversed causality). For example, if raters believe strongly in the success of an intervention and that intervention proves to be successful in the trial, they will favor assessment of a good outcome in the intervention arm and will judge treatment allocation correctly significantly more often than chance. Second, patients in IMS III were randomized in a 2:1 fashion. We assumed that the assessors did not take the 2:1 randomization ratio into account when judging the patient’s treatment allocation. If the assessors took the randomization ratio into account, the expected percentage of correct indications of treatment allocation based on chance would be higher (i.e. 55.6; (intervention: ⅔ × ⅔) + (control: ⅓ × ⅓) = 5/9 = 0.556). However, it is unlikely that assessors took the 2:1 randomization rate into account as they guessed “intervention group” in 254/459 (55.3%) of the patients. If they indeed took the 2:1 randomization ratio into account, one would expect that the assessors guessed “intervention group” in 306/459 (66.7%) of the patients. Another limitation of this study is that we excluded patients who died prior to 90-day assessment and patients with missing data on guessed treatment allocation. Unblinding of patients who died before 90-day follow-up is not relevant in terms of outcome assessment as the last category on the mRS is objective (death) in contrast to the other six categories. We do not know why assessors did not guess or did not report the treatment allocation of some patients and whether there were unknown differences between this group and the included patients. Importantly, a bias associated with baseline characteristics, treatment allocation, and outcome is unlikely, because patients included in this study and patients with unknown guessed treatment allocation had similar characteristics (Table 1).

In conclusion, we provide evidence that assessors may not always be truly blinded for treatment allocation in clinical trials, and their guesses may be associated with outcome. Although causality between the assessors’ guess and patient outcome cannot be determined, future trials with subjective outcome should make efforts to ensure blinding and should report their blinding method and the success of blinding like the IMS III trial.

## Supplemental Material

sj-docx-1-wso-10.1177_17474930221131706 – Supplemental material for Blinding of outcome assessors and its association with outcome in a randomized open-label stroke trial Click here for additional data file. Supplemental material, sj-docx-1-wso-10.1177_17474930221131706 for Blinding of outcome assessors and its association with outcome in a randomized open-label stroke trial by Nadinda AM van der Ende, Bob Roozenbeek, Joseph P Broderick, Pooja Khatri, Hester F Lingsma and Diederik WJ Dippel in International Journal of Stroke
