# Supplementary material for: Blinding of outcome assessors and its association with outcome in a randomized open-label stroke trial
Source: Int J Stroke. 2022 Oct 19;18(5):562–8. doi: 10.1177/17474930221131706 (PMC10196921; doi:10.1177/17474930221131706)
Supplement: sj-docx-1-wso-10.1177_17474930221131706 – Supplemental material for Blinding of outcome assessors and its association with outcome in a randomized open-label stroke trial [file sj-docx-1-wso-10.1177_17474930221131706.docx]

**SUPPLEMENTAL MATERIAL ON:**

**Blinding of outcome assessors and its association with outcome in a randomized open-label stroke trial**

*Nadinda A.M. van der Ende, MD;^1,2^ Bob Roozenbeek, MD, PhD;^1,2^ Joseph P. Broderick, MD, PhD;^3^ Pooja Khatri, MD, PhD;^3^ Hester F. Lingsma, PhD;^4^ Diederik W.J. Dippel, MD, PhD;^1^ on behalf of the IMS III Investigators*

Departments of Neurology^1^, Radiology and Nuclear Medicine^2^, Public Health^4^, Erasmus MC University Medical Center, Rotterdam, the Netherlands;

Department of Neurology and Rehabilitation Medicine^3^, University of Cincinnati Gardner Neuroscience Institute, University of Cincinnati Academic Health Center, OH, the United States of America

**Supplemental Table I.** Blindedness Questionnaire.

| 1 | As the blinded study investigator, into which cohort do you think the subject was randomized? | | 􀂅 IV / IA cohort | | 􀂅 IV only |
| --- | --- | --- | --- | --- | --- |
| 2 | How sure are you of this answer? | | 􀂅 Very sure  􀂅 Somewhat sure  􀂅 Not sure at all (it’s a guess) | | |
| 3 | Please check each item to indicate what your guess is based upon. | Improvement in symptoms | 􀂅 No | 􀂅 Yes | |
| 4 |  | Lack of improvement in symptoms | 􀂅 No | 􀂅 Yes | |
| 5 |  | Adverse effects of study treatment | 􀂅 No | 􀂅 Yes | |
| 6 |  | Lack of adverse effects of study treatment | 􀂅 No | 􀂅 Yes | |
| 7 |  | Other reasons (specify below) | 􀂅 No | 􀂅 Yes | |
| 8 |  | Specify, if other: |  | | |
| General comments: | | | | | |
